# Accurate Estimation of Running Temporal Parameters Using Foot-Worn Inertial Sensors

**DOI:** 10.3389/fphys.2018.00610

**Published:** 2018-06-12

**Authors:** Mathieu Falbriard, Frédéric Meyer, Benoit Mariani, Grégoire P. Millet, Kamiar Aminian

**Affiliations:** ^1^Laboratory of Movement Analysis and Measurement, École Polytechnique Fédérale de Lausanne, Lausanne, Switzerland; ^2^Institute of Sport Sciences, University of Lausanne, Lausanne, Switzerland; ^3^Gait Up S.A., Lausanne, Switzerland

**Keywords:** running, inertial measurement unit (IMU), validation study, temporal parameters, contact time

## Abstract

The aim of this study was to assess the performance of different kinematic features measured by foot-worn inertial sensors for detecting running gait temporal events (e.g., initial contact, terminal contact) in order to estimate inner-stride phases duration (e.g., contact time, flight time, swing time, step time). Forty-one healthy adults ran multiple trials on an instrumented treadmill while wearing one inertial measurement unit on the dorsum of each foot. Different algorithms for the detection of initial contact and terminal contact were proposed, evaluated and compared with a reference-threshold on the vertical ground reaction force. The minimum of the pitch angular velocity within the first and second half of a mid-swing to mid-swing cycle were identified as the most precise features for initial and terminal contact detection with an inter-trial median ± IQR precision of 2 ± 1 ms and 4 ± 2 ms respectively. Using these initial and terminal contact features, this study showed that the ground contact time, flight time, step and swing time can be estimated with an inter-trial median ± IQR bias less than 12 ± 10 ms and the a precision less than 4 ± 3 ms. Finally, this study showed that the running speed can significantly affect the biases of the estimations, suggesting that a speed-dependent correction should be applied to improve the system’s accuracy.

## Introduction

In running, two temporal events (initial contact or touchdown and terminal contact or toe-off) need to be detected in order to extract the main temporal parameters of each step: cadence, contact time, flight phase duration, and swing phase duration. Initial contact (IC) is defined as the time instant when the foot initiates contact with the ground at landing. Terminal contact (TC) corresponds to the end of the pushing phase, when the foot ends contact with the ground. The intrinsic relationships between the different inner-stride temporal parameters and running speed, shoe configuration, running economy, running performance, injury risks have been widely investigated. Therefore, an accurate detection of IC and TC are paramount.

In the literature, the majority of studies that investigated temporal parameters in running have used force plates, contact mats or high speed cameras as reference measurement system (Viitasalo et al., 1997; Garcia-Lopez et al., 2005; Leitch et al., 2011; Ogueta-Alday et al., 2013; Handsaker et al., 2016). Although force plates are accepted as state-of-the-art systems for temporal events detection in running, they suffer from several limitations. In fact, the detection timing of IC and TC on the vertical ground reaction force depends on the filtering method and on the detection threshold used (Cronin and Rumpf, 2014). Moreover, their lack of portability and their setup complexity restrict their use for in-laboratory experiments, which is a major drawback given the in-field nature of the running activity.

Thanks to the recent improvements in MEMS inertial sensors, their low production cost, their decrease in weight and size and their ability to measure kinematics over large periods of time, inertial sensors are now widely accepted systems to analyze human locomotion. In fact, studies on gait analysis have shown that inertial measurement units (IMUs), when used with state-of-the-art algorithms, can reliably fill the gap between subjective observational analysis and bulky in-laboratory installations (Mariani et al., 2012, 2013). In running, inertial sensors have predominantly been used to detect inner-stride temporal events and derive temporal parameters estimations from them. Some studies have used IMUs on the upper body (Bergamini et al., 2012; Norris et al., 2014), other focused on the shank/tibia segments (Mercer et al., 2003; Crowell et al., 2010; McGrath et al., 2012) and some used foot-worn IMUs (Strohrmann et al., 2011; Chapman et al., 2012; Lee et al., 2015; Reenalda et al., 2016; Brahms, 2017). However to the authors’ knowledge, only a few studies have reported on the validity of their algorithms when compared with state-of-the-art reference system. In Ammann et al. (2016), CT estimations were compared between shoe laces worn IMUs and a high-speed video camera for 132 steps of 12 athletes at running speeds within 22.3 ± 5.8 km/h. Because data processing was done by a proprietary software, the algorithms used to estimate CT were not described in the methods. In Weyand et al. (2001) the authors used acceleration peak from a foot-worn accelerometer to detect IC and TC and compared their estimation of CT with a treadmill-mounted force plate. The exact method used to detect IC and TC is not documented in this study and only the bias (mean ± STD) of the 165 trials is provided in the results. There is therefore, no information about the precision of the proposed system. For all other methods, where no validation was reported, there is no evidence that the parameters measured are within an acceptable error range and that this error range does not change with the running conditions.

Therefore the aim of the present study was to investigate different algorithms to detect IC and TC from different features measured by foot-worn IMU kinematic signals, and estimate the main inner-stride temporal parameters. The performance metrics (bias and precision) of each algorithm were assessed in comparison with a reference system (instrumented force plate treadmill), that allowed a validation of inner-stride temporal parameters over a high number of steps and a large range of running speeds.

## Materials and Methods

### Measurement Protocol

In total, 41 healthy adults (13 females and 28 males, age 29 ± 6 years, weight 70 ± 10 kg, height 174 ± 8 cm, running weekly 2.1 ± 1 h, 11 being affiliated to a running club) running at least once a week and without any symptomatic musculoskeletal injuries volunteered to participate to this study. The study was approved by the local ethic committee (CCER-VD 2015-00006), was conducted according to the declaration of Helsinki, and written informed consent was obtained from all the participants prior to the measurements. Each participant was asked to run multiple trials of 30 s each, wearing their usual shoes, on an instrumented treadmill, starting at 8 km/h and increasing by 2 km/h up to their maximum speed. A 6 min familiarization period (Lavcanska et al., 2005) was carried out on the treadmill and served as warm-up for the participants. The participants were free to decide on the rest duration in-between the trials.

### Wearable Device and Temporal Features Estimation

#### IMU Based System

One inertial measurement unit (IMU) (Physilog 4, Gait Up, Switzerland, weight: 19 g, size: 50 × 37 × 9.2 mm) was worn on the dorsum of each foot and measured both 3D acceleration and 3D angular velocity at 500 Hz. Each IMU was affixed to the foot using an adhesive strap around the shoe. The range of the accelerometer was set to ±16 g and ±2000°/s for the gyroscope.

#### Functional Calibration

**FIGURE 1:**
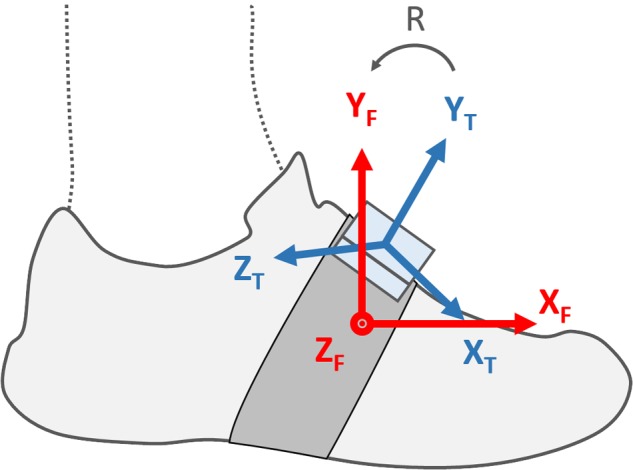
Shows both the technical frame of the foot-worn IMU (X_T_, Y_T_, Z_T_) and the functional frame of the foot (X_F_, Y_F_, Z_F_). The 3 by 3 rotation matrix R aligns the IMU’s technical frame with the functional frame of the foot.

In order to use single axes of the inertial sensors in a meaningful and reproducible manner, we designed a functional calibration method to automatically align the technical frame of the foot-worn IMUs with the functional frame of the foot. The functional frame of the foot was defined as in **Figure 1**: the origin is at the base of the second metatarsal bone, Y_F_ is orthogonal to the horizontal plane defined by the ground surface, X_F_ lies on the horizontal plane projection of the line joining the center of the calcaneus bone and the head of the second metatarsal bone, pointing distally, and Z_F_ is orthogonal to the X_F_Y_F_ plane pointing to the right-hand side of the subject. The functional calibration process requires static standing periods in order to align Y_T_ with Y_F_ using the gravitational acceleration measured by the IMU. Then, using the hypothesis that most of the foot’s angular rotations occur along the Z_F_ axis while running, we used Principal Component Analysis to find the rotation angle around the Z_T_ axis which aligns Z_T_ with Z_F_. Finally, X_T_ is the result of the cross-product <Z_T_, X_T_>.

#### Gait Cycle Detection

Using the cyclic nature of the running movement, an algorithm was designed to segment a complete trial into mid-swing to mid-swing cycles. Following previous work on gait analysis (Aminian et al., 2002; Sabatini et al., 2005), we hypothesized that the pitch angular velocity (ω_p_) of the foot is maximum at mid-swing. To enhance and detect the mid-swing peak, a 2nd-order Butterworth low-pass filter was designed with an adaptive cut-off frequency. The cut-off frequency was set at 60% of the stride frequency estimated using an auto-correlation method over a 5 s sliding window. This adaptive filtering method was used to cope with the range of running speeds used in this study. The length of the sliding window (5 s) was selected empirically and based on our observations of the signals.

#### Temporal Features Detection

**FIGURE 2:**
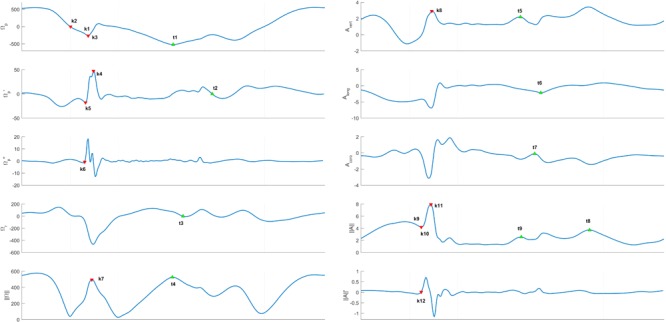
Features used on the kinematic signals recorded by the foot-worn inertial sensors. IC candidates are identified by kj with j ∈{1 … 12} and TC candidates are identified by tj with j ∈ {1 … 9}. The vertical gray dashed lines show the limits of the detection zones for IC and TC candidates. The signals showed in this figure belong to the same step and are represented during one mid-swing to mid-swing cycle.

The estimation of inner-stride phases relies on two main temporal events: initial and terminal contact. The initial contact (IC) event corresponds to the time instant when the foot initiates contact with the ground at landing. The terminal contact (TC) event, also known as toe-off, corresponds to the end of the pushing phase when the toes terminates contact with the ground. For each cycle, we identified kinematic features that seemed to be valid candidates to detect IC and TC. Such features varied from global maximum (MAX), local maximum (MAX_loc_), global minimum (MIN), local minimum (MIN_loc_) and zero crossing (ZeroX) time samples and were detected on the following signals: the pitch angular velocity (ω_p_: angular velocity around Z_F_), the pitch angular acceleration (ω’_p_), the pitch angular jerk or first derivative of the pitch angular acceleration (ω”_p_), the roll angular velocity (ω_r_: angular velocity around X_F_), the norm of the angular velocity (||ω||), the vertical axis acceleration (A_vert_: acceleration along Y_F_), the longitudinal axis of the acceleration (A_long_: acceleration along X_F_), the coronal axis acceleration (A_coro_: acceleration along Z_F_), the norm of the acceleration (||A||) and the first derivative of the acceleration norm or jerk (||A||). In some cases, an empirically chosen threshold was also used to improve the feature detection (e.g., < -100°/s). All these detection rules are detailed in **Table 1** and illustrated in **Figure 2**. Prior to the detection, the acceleration and angular velocity signals were filtered using a 2nd-order low-pass Butterworth filter (fc = 30 Hz) to minimize the influence of the IMU fixation artifacts and a temporal estimation of mid-stance was carried out for each gait cycle in order to separate the detection zones for IC and TC. The detection zone for IC was set as the period between the first zero-crossing of the pitch angular velocity (ω_p_) and mid-stance. For TC, the detection zone was set as the period between mid-stance and the last zero-crossing of the pitch angular velocity. Mid-stance was set as the time instant when the angular velocity norm (||ω||) is minimum within the 30–45% time-range of each mid-swing to mid-swing cycle. Finally, the IC and TC events of left and right foot steps were combined in order to estimate for each step i the ground contact time (CT), the flight time (FLT), the swing time (SWT) and the step time (SPT) using the following relations:

**Table 1:** Summary of the features used on the inertial sensors signals to detect initial contact (Ic) and terminal contact (Tc).

Detection zone	Feature			Description
	Signal	Rule	Label	
Initial contact (IC)	ω_p_	MIN	k1	Minimum of the pitch angular velocity
		ZeroX	k2	First zero-crossing of the pitch angular velocity
		MIN_loc_ < -100 °/s	k3	First local minimum smaller than 100°/s on the pitch angular velocity
	ω’_p_	MAX	k4	Maximum of the pitch angular acceleration
		MIN before k4	k5	Minimum of the pitch angular acceleration before k4
	ω”_p_	ZeroX	k6	Last zero-crossing of the pitch angular jerk before k4
	||ω||	MAX	k7	Maximum on the angular velocity norm
	A_vert_	MAX	k8	Maximum of the vertical acceleration
	||A||	MAX	k9	Maximum of the acceleration norm
		MIN before k9	k10	Minimum of the acceleration norm before k9
		MIN_loc_	k11	First local minimum of the acceleration norm
	||A||’	ZeroX	k12	Last zero-crossing of the jerk
Terminal contact (TC)	ω_p_	MIN	t1	Minimum of the pitch angular velocity
	ω’_p_	ZeroX after t1	t2	First zero-crossing of the pitch angular acceleration after t1
	ω_r_	ZeroX after t1	t3	First zero-crossing of the roll angular velocity after t1
	||ω||	MAX	t4	Maximum of the angular velocity norm
	A_vert_	MAX_loc_ after t1	t5	First local maximum of the vertical acceleration after t1
	A_long_	MIN	t6	Minimum of the longitudinal acceleration
	A_coro_	MAX_loc_ after t1	t7	First local maximum of the coronal acceleration after t1
	||A||	MAX	t8	Maximum of the acceleration norm
		MAX_loc_ after t1	t9	First local maximum of the acceleration norm after t1

*IC candidates are identified by kj with j ∈{1.. 12} and TC candidates are identified by tj with j ∈{1.. 9}. The features presented in this table were used in the respective detection zone of IC and TC.*

(1) $$\begin{array}{c} {\mathrm{CT}}_{i}\;=\;{\mathrm{TC}}_{i}\;-\;{\mathrm{IC}}_{i} \end{array}$$

(2) $$\begin{array}{c} {\mathrm{FLT}}_{i\;}=\;{\mathrm{IC}}_{i}{\;}_{+}{\;}_{1}\;-\;{\mathrm{TC}}_{i} \end{array}$$

(3) $$\begin{array}{c} {\mathrm{SWT}}_{i}\;=\;{\mathrm{IC}}_{i}{\;}_{+}{\;}_{2}\;-\;{\mathrm{TC}}_{i} \end{array}$$

(4) $$\begin{array}{c} {\mathrm{SPT}}_{i}\;=\;{\mathrm{IC}}_{i}{\;}_{+}{\;}_{1}-\;{\mathrm{IC}}_{i} \end{array}$$

### Reference System and Temporal Features

#### Force Plate

This study used an instrumented treadmill (T-170-FMT, Arsalis, Belgium) sampling at 1000 Hz as reference system for the validation. The force plate system and the inertial sensors were electronically synchronized using a 5 V pulse triggered manually and recorded on each system while IMUs were synchronized with each other’s using radio frequencies. To reduce the noise inherent to the treadmill’s vibrations, we first applied, on the vertical ground reaction force (GRF) signal, a 2nd-order stop-band Butterworth filter with edge frequencies set to 25 and 65 Hz. The filter configuration was chosen empirically to obtain a satisfactory reduction of the oscillations observed during flight phases (i.e., subject not in contact with the treadmill) while minimizing its widening effect during ground contact timeS.

#### Temporal Features Detection

IC and TC events were detected using a threshold on the filtered vertical GRF signal, setting the first threshold-crossing occurrence as IC and the second as TC for each step. As previous studies (Weyand et al., 2001; Cronin and Rumpf, 2014) used different reference thresholds, we have decided to investigate the effect of eight reference thresholds on the validation results. Four thresholds were set to 20, 30, 40, and 50 N, independently of the subjects’ body weight (BW) and four others were set to 3, 5, 7, and 9 %BW. Finally, we combined IC and TC events to find the reference inner-stride phases durations (CT, FLT, SWT, and SPT) as in Equations 1–4.

### Statistical Analysis and Error Estimation

In order to avoid developing algorithms that over-fits our data set and would therefore bias the results, first 10 subjects were randomly selected and dedicated to the development set while the remaining subjects were only used as the validation set. The design of the algorithms described in Section “Wearable Device and Temporal Features Estimation” was conducted using solely the data from the development set. No algorithms debugging was done over signals from the validation set.

To evaluate the error of the proposed system against the reference force plate, we computed for each temporal feature, the bias (intra-trial mean) and precision (intra-trial STD) for all steps within a trial. We then combined the results from each trial and computed the median and IQR of both the bias and precision over all trials. These two steps resulted in four inter-trial statistics per temporal feature for both sets (development and validation sets): b_μ_ is the inter-trials median bias, b_σ_ is the inter-trials IQR of the bias, σ_μ_ is the inter-trials median precision and σ_σ_ is the inter-trials IQR of the precision. Note that we have used the median and IQR functions for the inter-trial statistics as the intra-trial bias and precision were not normally distributed.

A similar method was used for the inner-stride phases. However, to avoid having a large number of candidates for each parameter (12 IC candidates ^∗^ 9 TC candidates = 108 possible pairs of candidates for each phase estimation), we have decided to keep only the three most precise candidates for IC, the three most precise candidates for TC and to combine them into 9 pairs of estimates for CT, FLT, SWT, and SPT. Then, similarly, the inter-trials bias (b_μ_, b_σ_) and the inter-trial precision (σ_μ_, σ_σ_) were evaluated. Precision (i.e., intra-trial STD) was chosen as selection criteria for IC and TC candidates as it informs about the range of random errors made by the system among the steps of a trial. The bias, however can potentially be decreased using an appropriate model of the errors.

To investigate if the speed affects the intra-trial bias of the IC and TC candidates, we used the Kruskal–Wallis test with a significance level of 0.05. We preferred this non-parametric test to the one-way ANOVA because the Lilliefors test rejected, in most cases, the hypothesis that the intra-trial bias were normally distributed among the running speeds. Consequently, in this study, the null hypothesis was accepted only if the rank of the biases were equal among the running speeds. The same hypothesis has also been tested on the precision. Note that this test was applied on the complete data set (development and validation set) as there was no speed-depend adaptations of our detection algorithms.

Finally, we used Bland-Altman plots and the best linear fit, in the least squares sense, to show the trend in the CT estimation errors on the development set. Finding the best linear fit on the development set, allows to further use the linear coefficients to correct the inter-steps errors in the validation set. The inter-steps errors refers to the error of all steps within a group, independently of the trial they belong to. The inter-steps bias is defined as the mean error of all steps and the inter-steps precision as the STD of the error of all steps.

## Results

### Temporal Events Detection

Out of the 41 participants, 35 were kept for the evaluation of the proposed system. Within the 6 participants removed, 2 were removed because the data loss rate was above 20% and 4 were removed because of calibration errors of the systems. The results for the development set and the validation set were computed from 10 subjects with 59 trials (4836 steps) and 25 subjects with 146 trials (12092 steps), respectively. Trials with running speed at 8 km/h were removed due to the presence of steps with double support for some subjects that makes the detection of IC and TO impossible with the GRF of the reference system. The minimum number of steps per trial was 67 and the maximum number of steps per trial was 105 given that the running speed recorded ranged from 10 to 20 km/h. **Figure 2** illustrates the features used to detect IC and TC with the vertical gray dashed lines showing the limits of the detection zones for IC and TC candidates. The signals showed in **Figure 2** belong to the same step and are represented during one mid-swing to mid-swing cycle.

**Table 2** summarizes the IC and TC events detection error for development and validation sets, and for each kinematics feature candidate (kj and tj) extracted by applying the specific detection rule on the kinematics signal. The results are obtained by using the reference value estimated with a threshold at 7 %BW on the vertical GRF. The differences shown in the table were computed such that a positive difference indicates that the event was detected later in the signal than the reference. The three most precise IC candidates (median ± IQR) with respect to the results from the validation set are: k_1_ (2 ± 1 ms), k_3_ (2 ± 1 ms) and k_8_ (3 ± 2 ms). The three most precise TC candidates (median ± IQR) with respect to the results from the validation set are: t_1_ (4 ± 2 ms), t_4_ (4 ± 2 ms) and t_5_ (4 ± 2 ms). One TC candidate shows a noticeably lower inter-trial bias IQR: t_5_ with b_σ_ = 7 ms.

**Table 2:** List of time differences for all the IC and TO candidates, computed over 4836 and 12092 steps for the development set and the validation set, respectively.

	Feature			Development set (*N* = 59) errors when threshold at 7%BW is used on vertical GRF (ms)				Validation set (*N* = 146) errors when threshold at 7%BW is used on vertical GRF (ms)			
	Signal	Rule	Label	b_μ_	b_σ_	σ_μ_	σ_σ_	b_μ_	b_σ_	σ_μ_	σ_σ_
Initial contact (IC)	ω_p_	MIN	k1	11	14	2	1	11	10	2	1
		ZeroX	k2	–30	11	6	3	–29	11	6	2
		MIN_loc_ < -100°/s	k3	11	14	2	1	11	10	2	1
	ω’_p_	MAX	k4	22	20	3	2	23	15	4	2
		MIN before k4	k5	–5	7	3	4	–4	7	4	4
	ω”_p_	ZeroX	k6	–3	11	2	3	–2	8	3	3
	||ω||	MAX	k7	14	4	3	2	14	5	4	2
	A_vert_	MAX	k8	19	13	3	2	20	13	3	2
	||A||	MAX	k9	19	18	3	3	17	17	3	3
		MIN before k9	k10	1	19	3	5	0	13	5	6
		MIN_loc_	k11	6	19	7	5	4	13	7	5
	||A||’	ZeroX	k12	2	17	2	4	2	13	3	4
Terminal contact (TC)	ω_p_	MIN	t1	–24	14	3	2	–21	13	4	2
	ω’_p_	ZeroX after t1	t2	31	18	10	13	29	17	9	10
	ω_r_	ZeroX after t1	t3	33	24	13	39	39	33	14	25
	||ω||	MAX	t4	–22	14	3	2	–18	13	4	2
	A_vert_	MAX_loc_ after t1	t5	–7	8	4	3	–4	7	4	2
	A_long_	MIN	t6	20	18	5	9	18	15	6	7
	A_coro_	MAX_loc_ after t1	t7	–2	14	21	9	1	11	22	9
	||A||	MAX	t8	33	38	24	28	37	57	22	40
		MAX_loc_ after t1	t9	–3	11	4	2	0	13	5	6

*Time differences are expressed in milliseconds (ms). The reference system used in this table is the vertical GRF with a threshold set at 7% BW. IC candidates are identified by kj with j ∈{1.. 12} and TC candidates are identified by tj with j ∈{1.. 9}. “b” and “σ” are the abbreviations for accuracy (intra-trial mean error) and precision (intra-trial STD of the error), respectively, while suffix “μ” and “σ” represent the median and the IQR over all the trials.*

**FIGURE 3:**
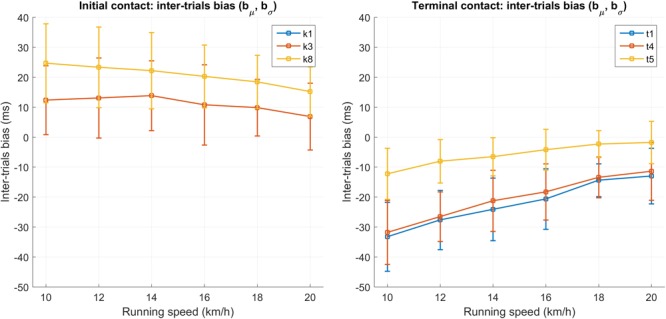
Initial contact **(left graph)** and terminal contact **(right graph)** inter-trials bias for the features (k_1_, k_3_, k_8_) and (t_1_, t_4_, t_5_), respectively. The graph was computed using the complete data set (development set and validation set) and using the reference threshold on the vertical GRF at 7 %BW. Each group of speed contains *N* = 35 trials except the 20 km/h group where *N* = 30.

**Figure 3** shows the influence of the running speed on the IC and TC inter-trials bias for the features (k_1_, k_3_, k_8_) and (t_1_, t_4_, t_5_). The graph was generated using the complete data set (development and validation set) as it is solely used for visualization purpose. When the trials are grouped according to the running speed, the Kruskal–Wallis test applied on the biases shows that the running speed significantly affects the biases in k_8_ (*p* = 0.001), t_1_ (*p* < 0.001), t_4_ (*p* < 0.001), t_5_ (*p* < 0.001) and precision in t_1_ (*p* < 0.001), t_4_ (*p* = 0.014) and t_5_ (*p* < 0.001).

### Inner-Stride Phases Estimation

**Table 3** lists absolute and relative errors obtained for the estimations of CT, on the validation set, when compared with the force plate estimation found using the reference threshold at 7 %BW. The bias and precision obtained when comparing the other force plate thresholds with the 7%BW reference threshold are also listed at the end of **Table 3**.

**Table 3:** List of the duration differences for CT estimation in the validation set (*N* = 146 trials, 12092 steps) when compared to the force plate estimation using the reference threshold set at 7 %BW.

Features		CT errors when compared with reference at 7 %BW (ms)				CT errors when compared with reference at 7 %BW (%)			
IC	TC	b_μ_	b_σ_	σ_μ_	σ_σ_	b_μ_	b_σ_	σ_μ_	σ_σ_
k1	t1	–30	17	4	2	–13.8	5.5	1.8	0.9
k1	t4	–27	17	4	2	–12.9	5.5	1.9	1.2
k1	t5	–15	12	5	3	–7.1	5.0	2.1	1.0
k3	t1	–30	18	4	2	–13.8	5.6	1.8	1.0
k3	t4	–27	17	4	3	–12.9	5.5	1.9	1.4
k3	t5	–15	12	5	3	–7.1	5.2	2.2	1.1
k8	t1	–38	21	5	3	–18.1	6.0	2.1	1.0
k8	t4	–35	21	5	3	–17.4	6.1	2.2	1.3
k8	t5	–23	15	5	3	–10.8	5.5	2.2	1.3
20 N		8	6	3	1	4.0	2.2	1.3	0.9
30 N		5	4	2	1	2.2	1.6	1.0	0.6
40 N		2	3	1	1	0.9	1.2	0.6	0.5
50 N		0	2	1	1	–0.1	1.1	0.4	0.3
3 %BW		9	5	3	2	3.9	1.7	1.3	0.9
5 %BW		4	2	2	1	1.7	0.6	0.8	0.5
9 %BW		–3	2	2	1	–1.4	0.5	0.7	0.4

*The first nine rows show the estimation errors of the three most precise candidates for IC and TO detection arranged as pairs while the last seven rows show the difference observed when using other reference thresholds on the vertical GRF signal. “b” and “σ” are the abbreviations for bias (intra-trial mean error) and precision (intra-trial STD of the error), respectively, while subscript characters μ and σ represent the median and the IQR over all the trials in the validation set.*

The most precise pair of IC and TC candidates for CT was (k_1_, t_1_) with an inter-trial median ± IQR precision of 4 ± 2 ms or 1.8 ± 0.9%. CT estimators (k_1_, t_5_) and (k_3_, t_5_) both have the lowest absolute inter-trial IQR of the biases (b_σ_ = 12 ms) while (k_1_, t_5_) has the lowest IQR in relative values (b_σ_ = 5.0%). The reference values observed in this study ranged from 132 to 354 ms for CT, from 29 to 238 ms for FLT, from 367 to 613 ms for SWT and from 254 to 435 ms for SPT. **Table 4** shows the relative and absolute errors for FLT, SWT, and SPT estimations for both (k_1_, t_1_), (k_1_, t_5_) and (k_3_, t_5_) pairs.

**Table 4:** Flight phase duration (FLT), swing phase duration (SWT) and step time duration (SPT) estimations errors for the (k_1_, t_1_), (k_1_, t_5_) and (k_3_, t_5_) candidates when a reference threshold at 7 %BW is used on the vertical GRF.

Parameter	Estimator	Absolute errors when compared with reference threshold at 7 %BW (ms)				Relative errors when compared with reference threshold at 7 %BW (%)			
		b_μ_	b_σ_	σ_μ_	σ_σ_	b_μ_	b_σ_	σ_μ_	σ_σ_
FLT	(k1, t1)	30	17	4	3	22.8	17.2	4.0	2.8
	(k1, t5)	15	12	5	3	10.7	10.7	3.7	2.7
	(k3, t5)	15	12	5	3	10.7	10.7	3.9	2.6
SWT	(k1, t1)	30	17	4	2	6.3	3.7	0.9	0.4
	(k1, t5)	15	12	5	3	3.2	2.6	1.0	0.6
	(k3, t5)	15	12	5	3	3.2	2.6	1.0	0.6
SPT	(k1, t1)	0	0	3	2	0.0	0.0	0.8	0.5
	(k1, t5)	0	0	3	2	0.0	0.0	0.8	0.5
	(k3, t5)	0	0	3	2	0.0	0.0	0.8	0.5

*The results were computed from the data in the validation set (*N* = 146 trials, 12092 steps). “b” and “σ” are the abbreviations for bias (intra-trial mean error) and precision (intra-trial STD of the error), respectively, while subscript characters μ and σ represent the median and the IQR over all the trials in the validation set.*

**FIGURE 4:**
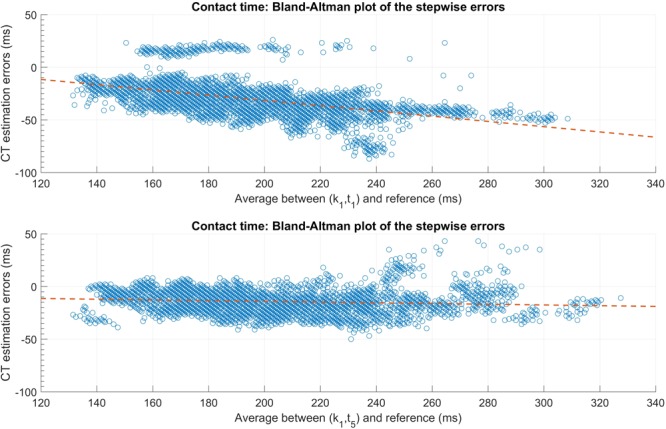
Bland-Altman plot of the ground contact time (CT) estimation errors for the (k_1_, t_1_) **(top graph)** and (k_1_, t_5_) **(bottom graph)** candidates. The error is measured on all the steps of the development set (*N* = 4836). The orange dashed line represent the best linear fit according to the least square method.

Finally, **Figure 4** shows the Bland-Altman plot for the CT estimation of the (k_1_, t_1_) and (k_1_, t_5_) estimators. The orange dashed line represent the best linear fit according to the least squares method. These graphs were computed using all the steps in the development set (*N* = 4836), independently of the trials.

## Discussion

In this study we proposed, evaluated and compared how different algorithms based foot-worn IMU kinematic features performed in detecting IC and TC during running and in estimating the main inner-stride temporal parameters: CT, FLT, SWT, and SPT. The errors (displayed in **Table 2**) show that the bias and precision for IC and TC could reach very low values depending on the kinematic features used. Therefore by considering the most performant kinematic features an accurate and precise estimation of inner-stride temporal parameters was proposed and validated against a force plate as reference system.

**Table 3** shows that, the three most precise IC candidates (k_1_, k_3_ and k_8_) and TC candidates (t_1_, t_4_, and t_5_) can be combined to provide a precise estimation of ground contact time (CT). The most precise pair of features obtained from the two minimums of pitch angular velocity in IC and TC detection zones (k_1_, t_1_) had an inter-trials median ± IQR precision of 4 ± 2 ms (1.8 ± 0.9%). However the accuracy of the t_1_ candidate is speed dependent (*p* < 0.001). This explains the relatively high inter-trial IQR of the biases (b_σ_ = 17 ms) of CT for the (k_1_, t_1_) candidate. In **Figure 3**, the median of the biases for the t_1_ (as well for t_4_ and t_5)_ seem to linearly decrease as the speed increases. However, even though the Kruskal–Wallis test shows that speed also affect t_5_ (*p* < 0.001), the range of the median biases is approximately two times shorter for t_5_ (10 ms) than for t_1_ (21 ms).

To reduce the effect of the running speed on the bias, the minimums of pitch angular velocity in IC zone and the maximum of vertical acceleration in TC zone, i.e., (k_1_, t_5_) candidate can be used. Although it is slightly less precise on the detection of CT, the results in **Table 4** show better results in the estimation of FLT for both the accuracy and precision. Given that the CT decreases as speed increase, a measure of the CT itself already contains information about the running speed. Therefore, using the coefficients from the best linear fit (development set data) showed on the Bland-Altman plots in **Figure 4**, the validation set inter-trials median ± IQR bias decreased to -2 ± 14 ms (-1 ± 6.2%) and 1 ± 10 ms (0.3 ± 4.9%) for the (k1, t1) and the (k1, t5) pairs, respectively. For both the (k1, t1) and the (k1, t5) candidates, the precision did not change after the aforementioned correction. Note that the outliers observed on the top graph of **Figure 4** correspond to the detection errors of the t_1_ feature due to a second minimum happening later in the pitch angular velocity signal.

Moreover, **Table 2** reveals that the most precise features for IC detection were found on the measurements from a single axis of the IMUs (k_1_, k_3_, and k_8_). This observation emphasizes on the importance of the functional calibration which aligns the technical frame of the inertial sensors with the biomechanically meaningful axes of the foot.

**Table 2** also shows that, in general, the kinematic features used in this study tend to better detect IC than TC. Considering that the IC event comes with a landing impact, while no abrupt variation in the foot’s motion occurs at TC, the odds of missing the exact instant of TC are higher. Moreover, the vertical force applied by the foot on the ground decreases drastically at the end of the CT although foot is still in contact with the ground leading to a potentially early detection of TC. Similar observations were reported by Weyand et al. (2001). In fact, we observed that the 3%BW detection threshold showed a bias (b_μ_ ± b_σ_) of -2 ± 2 ms and 7 ± 4 ms for IC and TC when compared to the 7%BW reference threshold. For both IC and TC, the bias was the highest when compared to a force threshold set at 20N. These results show that the detection accuracy of the force plate for TC, is more sensitive to the variations in the reference threshold than IC.

Lastly, the inter-step errors of the k_1_ feature seem to follow a bimodal distribution when including all step of the validation set, independently of the trials (*N* = 12092 steps). This implies that there might be an additional source of variance other than running speed that affects the detection of IC. Because the k_1_ feature is based on the angular velocity of the foot at landing, we assume that the type of foot-strike employed (fore-foot strike or rear-foot strike) could also introduce an error in the detection of IC. Further study would be required to evaluate how foot-strike angle influences detection accuracy and precision of temporal events during running. In addition, determining the applicability of the algorithms developed for level running in this study to uphill or downhill running would also need further study.

This study used a different method to express the CT errors than in Ammann et al. (2016). In the aforementioned study, the authors reported an inter-steps bias (*N* = 132 steps) of -1.9 ms (-1.3%) and a random error (95% confidence interval) of 17.4 ms (6.1%) for CT. The inter-steps bias and precision for the (k_1_, t_1_) pair showed comparable results. In fact, the validation set inter-steps bias (*N* = 12092 steps) was -2 ms (-0.5%) for CT, after applying the linear fit correction showed in the Bland-Altman plots in **Figure 4**. However, the inter-steps random error (95% confidence interval) was slightly higher (23 ms) for the (k1, t1) pair than in Ammann et al. (2016). This can be explained by the fact that t_1_ precision is affected by speed (*p* < 0.001) and that the range of speed in this study (10 – 20 km/h) is larger than in Ammann et al. (2016) (22.3 ± 5.8 km/h). In Weyand et al. (2001), the authors reported a bias (mean ± STD) of 14.6 ± 0.5% when computed over 165 trials. These results are in accordance with the biases showed in **Table 3**.

To the authors’ knowledge this study is the first to quantitatively demonstrate how, when using foot-worn IMUs in running, the choice of kinematic features affect the detection accuracy and precision of IC, TC and the inner-stride parameters derived from these two events. Consequently, it is important that researchers report on the methods applied to detect IC and TC events as it provides some information about the confidence interval of the measurements.

## Conclusion

This study aimed to validate, against a gold standard reference system, the performance of several algorithms using foot-worn inertial sensors to detect running gait temporal events and estimate inner-stride phases duration. The results highlighted the importance of suitable kinematic signals and features to avoid large errors in detecting initial and terminal contact. The two minimum values of the pitch angular velocity in the first half and second half of a mid-swing to mid-swing cycle provide the best estimation of IC and TC. Also the maximum value of vertical acceleration during the second half mid-swing to mid-swing cycle provides a good estimation of TC which is less dependent on running speed. Using these initial and terminal contact features, we showed that the ground contact time, flight time, step and swing time can be estimated with an inter-trial median ± IQR bias less than 15 ± 12 ms and the inter-trial median ± IQR precision less than 4 ± 3 ms. Running speed could have significant impact on the biases of the estimations and therefore the knowledge about the speed could improve the results. Further studies should investigate the effect of the foot-strike angle on the errors made by the features during initial contact.

## Author Contributions

MF, FM, BM, GM, and KA conceptualized the study design. MF and FM conducted the data collection. MF designed the algorithms and KA supervised the study. MF, FM, BM, GM, and KA contributed to the analysis and interpretation of the data. MF drafted the manuscript, all other authors revised it critically. All authors approved the final version, and agreed to be accountable for all aspects of this work.

## Conflict of Interest Statement

BM was employed by company Gait Up. The other authors declare that the research was conducted in the absence of any commercial or financial relationships that could be construed as a potential conflict of interest.
